# Global trends in immune mediated inflammatory diseases: Psoriasis, atopic dermatitis, multiple sclerosis, inflammatory bowel disease, and rheumatoid arthritis

**DOI:** 10.1016/j.jdin.2024.11.003

**Published:** 2024-11-26

**Authors:** Andrea Gelabert-Mora, Brenda Chiang, Ahnna Lee, Mahima Sinha, Katrina Abuabara

**Affiliations:** a Department of Dermatology, University of California–San Francisco, San Francisco, California; b San Juan Bautista School of Medicine, Caguas, Puerto Rico; c School of Public Health, University of California–Berkeley, Berkeley, California

**Keywords:** aging, atopic dermatitis, epidemiology, IMIDs, immune mediated inflammatory diseases, incidence, inflammatory bowel disease, multiple sclerosis, prevalence, psoriasis, rheumatoid arthritis

*To the Editor:* Immune-mediated inflammatory diseases (IMIDs) are a heterogeneous group of disorders characterized by chronic inflammation and sporadic flares.^1^ They include psoriasis, atopic dermatitis, multiple sclerosis, inflammatory bowel disease and rheumatoid arthritis, among others. Although traditionally characterized by organ system involvement, new molecular classification systems and response to targeted immunomodulatory treatments suggest commonalities across IMIDs that affect different organs.^2^^,^^3^

It has been postulated that IMIDs may share common environmental triggers, including diet, stress, pollution and microbes,^1^ but epidemiologic data on trends by age and sociodemographic factors are limited.^4^ We sought to describe and compare trends of the global and United States prevalence of IMIDs across age groups, sex, and sociodemographic index (SDI) over time.

Leveraging data from the Global Burden of Disease Study, a living database hosted by the Institute of Health Metrics Evaluation at the University of Washington,^5^ we calculated global and US prevalence of IMIDs with available data. These included atopic dermatitis, psoriasis, multiple sclerosis, inflammatory bowel disease, and rheumatoid arthritis. Additionally, we examined associations between low, medium, and high SDI countries, age categorized into 3 groups (<20 years, 20-54 years, and >55 years), sex, and calendar year using multivariable linear regression models.

The mean prevalence of aggregate IMIDs from 1990 to 2018 was 0.74% globally, affecting more than 230,000,000 individuals in 2018. In the United States, the overall mean prevalence of all IMIDs was 1.16%. Among high SDI countries, the global prevalence was 1.19%, as compared to 0.40% among low SDI countries, and 0.84% among females as compared to 0.64% among males globally (Table I).

**Table I:** Comparison of trends in immune-mediated inflammatory diseases (IMIDs) among United States and global models

Condition	Global prevalence	US prevalence
Any IMID	0.74%	1.16%
Atopic dermatitis	2.54%	3.56%
Inflammatory bowel disease	0.08%	0.25%
Multiple sclerosis	0.03%	0.11%
Psoriasis	0.74%	1.42%
Rheumatoid arthritis	0.31%	0.46%

Covariate	Global model			US model		
	Prevalence	Beta∗	95% confidence interval	Prevalence	Beta∗	95% confidence interval
Sex						
Male	0.64%	Ref	Ref	1.06%	Ref	Ref
Female	0.84%	0.0019	(0.00094, 0.0029)	1.26%	0.0021	(−0.0002, 0.0043)
Age						
<20	0.96%	0.0042	(0.0030, 0.0054)	1.56%	0.0079	(0.0051, 0.011)
20-54	0.54%	Ref	Ref	0.77%	Ref	Ref
>55	0.73%	0.0019	(0.0007, 0.0031)	1.14%	0.0037	(0.0010, 0.0065)
SDI						
Low	0.40%	−0.0025	(−0.0037, −0.0013)	N/A	N/A	N/A
Middle	0.64%	Ref	Ref	N/A	N/A	N/A
High	1.19%	0.0055	(0.0043, 0.0067)	N/A	N/A	N/A
Calendar year	N/A	−0.0000093	(−0.000068, 0.000049)	N/A	−0.000051	(−0.00019, 0.000083)

*SDI*, Sociodemographic index.

∗ Results from multivariable models predicting IMID prevalence. Betas represent the percent change for each covariate (ie, female sex is associated with 0.19% increase in IMID prevalence in the global model.

Globally, prevalence was highest among older adults (0.73% among those 55+ years) and among children and adolescents (0.96% among those <20 years) and was stable over time (Fig 1). Among IMIDs, atopic dermatitiswas most common, followed by psoriasis, then rheumatoid arthritis, inflammatory bowel disease, and multiple sclerosis (Table I). In a multivariable model, female sex, higher SDI, and age <20 or age ≥55 were associated with higher prevalence of IMIDs. In individual IMID models, age ≥55 years was significantly associated with increased prevalence of psoriasis, rheumatoid arthritis, multiple sclerosis, and inflammatory bowel disease both globally and in the United States.

**Fig 1:**
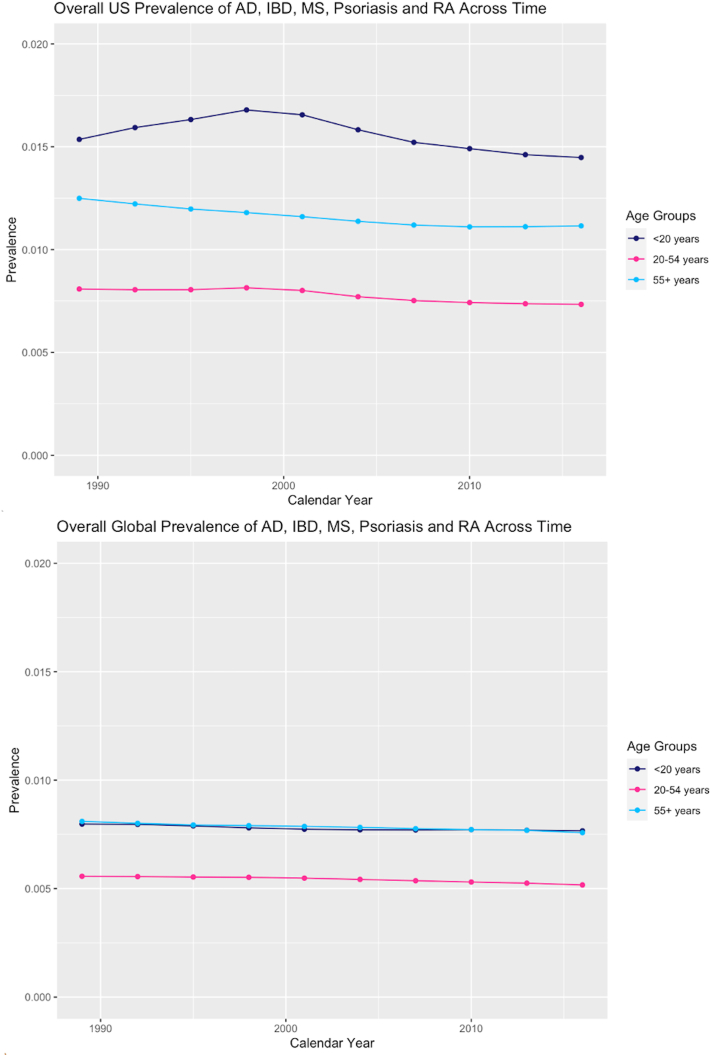
Overall global vs US prevalence of immune-mediated inflammatory diseases by age group across time. *AD*, Atopic dermatitis; *IBD*, inflammatory bowel disease; *MS*, multiple sclerosis; *RA*, rheumatoid arthritis.

Our results align with prior limited literature on IMID trends by sex and age, and highlight high rates among older adults, a relatively under-studied patient group. The mean US prevalence was higher across age groups compared to the global average, coinciding with an overall trend for higher prevalence of IMIDs in countries of higher SDI.^4^ Limitations of our study include a lack of disease severity measures and heterogeneous data collection methods (including data from censuses, population registries, insurance claims, hospital data, demographic surveillance systems, and other information portals), which are balanced by the benefit of estimates that are standardized globally through a Bayesian modeling process. In summary, female sex, high SDI, and youngest and oldest age groups are associated with higher IMIDs prevalence.

## Conflicts of interest

Dr Abuabara is a consultant and speaker and receives fees from Sanofi, Nektar, Amgen, TARGET RWE, and receives research grants from Pfizer and Cosmequite International SNC. Drs Gelabert-Mora, Lee, and Authors Sinha and Chaing have no conflicts of interest to declare.
